# Knowledge, attitudes, and practices regarding ventilator-associated pneumonia among family members of ICU patients

**DOI:** 10.3389/fpubh.2026.1997727

**Published:** 2026-10-30

**Authors:** Jian Zhou, Yonghong Shen, Jingbo Lu, Danyang Bai, Mengwen Shen, Huan Tang, Wei Zhang, Hui Liao, Ruiping Wang

**Affiliations:** 1 Intensive Care Unit, Yueyang Hospital of Integrated Traditional Chinese and Western Medicine, Shanghai University of Traditional Chinese Medicine, Shanghai, China; 2 Department of Nursing, Yueyang Hospital of Integrated Traditional Chinese and Western Medicine, Shanghai University of Traditional Chinese Medicine, Shanghai, China; 3 Department of Spine Surgery, Yueyang Hospital of Integrated Traditional Chinese and Western Medicine, Shanghai University of Traditional Chinese Medicine, Shanghai, China; 4 Department of Intensive Care Medicine, Affiliated Oriental Hospital of Tongji University, Shanghai, China; 5 Clinical Research Center, Affiliated Dermatology Hospital of Tongji University, Shanghai, China

**Keywords:** attitude, cross-sectional studies, family, health behavior, intensive care units, knowledge, pneumonia, ventilator-associated pneumonia

## Abstract

**Objective:**

Ventilator-associated pneumonia (VAP) remains a major complication of mechanical ventilation, while the role of family members in preventive care has been insufficiently explored. This study aimed to investigate the knowledge, attitudes, and practices (KAP) regarding VAP among family members of intensive care unit (ICU) patients.

**Methods:**

This cross-sectional study was conducted among family members of ICU patients receiving invasive mechanical ventilation at the Yueyang Hospital of Integrated Traditional Chinese and Western Medicine from July 1, 2025 to March 31, 2026. Participants were recruited using convenience sampling. Demographic characteristics and knowledge, attitude, and practice scores were collected and assessed using a self-designed structured questionnaire. The cutoff for good knowledge, positive attitudes, and proactive practices was defined as at least 70.0% of the corresponding total score. Descriptive analysis, multivariable regression, and structural equation modeling (SEM) were performed to evaluate factors associated with KAP outcomes and explore relationships among KAP domains.

**Results:**

A total of 406 participants were included, with 56.9% being female. The mean knowledge, attitude, and practice scores were 3.48 ± 5.26 (possible range: 0–24), 38.94 ± 6.62 (possible range: 10–50), and 19.33 ± 6.50 (possible range: 7–35), respectively. SEM showed direct effects of knowledge on attitude (*β* = 0.190, *p* < 0.001) and practice (*β* = 0.222, *p* < 0.001), as well as a direct effect of attitude on practice (*β* = 0.329, *p* < 0.001). Knowledge also indirectly affected practice through attitude (*β* = 0.063, *p* = 0.001).

**Conclusion:**

Family members of ICU patients generally held poor VAP-related knowledge, positive attitudes, and poor practices. Knowledge and attitudes were key determinants of preventive practices, with attitude mediating the knowledge–practice pathway. Structured family-oriented education and bedside participation protocols should be integrated into ICU care to improve feasible VAP-prevention behaviors.

## Introduction

Ventilator-associated pneumonia (VAP) remains a common and clinically important hospital-acquired infection among mechanically ventilated patients in intensive care units (ICUs) (1). VAP remains a critical patient safety and resource-utilization challenge worldwide, with persistently high incidence reported even in high-resource settings (2). Recent evidence indicates that approximately 30% of ICU patients receiving mechanical ventilation develop VAP (3), which substantially prolongs ventilator dependence and hospitalization, with reported increases of 12.3 days for mechanical ventilation, 12.7 days for ICU stay, and 16.1 days for total hospitalization (4). VAP imposes a substantial antimicrobial burden, accounting for approximately half of all antibiotic use in the ICU (5), and its long-term health impacts on survivors include impaired functional capacity, reduced muscle strength, decreased pulmonary function, and lower quality of life (6). Effective prevention and management rely on adherence to evidence-based guidelines and coordinated multi-modal interventions, such as ventilator care bundles, which have been shown to significantly reduce both VAP incidence and duration of mechanical ventilation (1).

Beyond clinical staff, family members of mechanically ventilated ICU patients also play distinctive roles in patient care. They often serve as communication bridges between patients and healthcare providers, particularly when patients are sedated or unable to express their needs, facilitating the transfer of information regarding medical history, preferences, and changes in condition (7). They also act as caregiving collaborators, supporting healthcare staff with repositioning, oral care assistance, and observation of clinical changes during bedside visits (8). In addition, family members can contribute to non-invasive preventive behaviors such as hand hygiene before patient contact, encouraging early mobilization or coughing exercises when appropriate, and promptly notifying staff of ventilator alarms or worsening symptoms (9). Given these multifaceted roles, the knowledge, attitudes, and practices of family members regarding VAP may directly influence the quality of bedside prevention and the effectiveness of ventilator care bundles, making their assessment a necessary complement to existing professional-focused research.

Knowledge, attitude, and practice (KAP) research provides a structured framework to understand and influence health-related behaviors. KAP surveys are widely used in public health to assess the cognitive, affective, and behavioral dimensions of disease prevention and to identify gaps between awareness and implementation (10, 11). Existing KAP-related studies on VAP prevention have mainly focused on ICU nurses, addressing oral care practices, knowledge of evidence-based preventive guidelines, and barriers to guideline adherence in clinical practice (12, 13). These studies demonstrate that even when attitudes toward VAP prevention are positive, knowledge gaps and organizational constraints may impede optimal practice. Meanwhile, family-centered ICU care has become an important component of contemporary critical care, recognizing family members not merely as visitors but as active participants in patient support, information sharing, communication, and selected aspects of bedside care (14, 15). Families’ involvement in patient care may therefore influence the effectiveness of VAP prevention strategies, yet their KAP regarding VAP remains poorly characterized.

Despite growing recognition of family engagement in ICUs, there is a paucity of research investigating the knowledge, attitudes, and practices of family members concerning VAP prevention. Accordingly, this study aims to systematically assess the KAP of family members of mechanically ventilated ICU patients.

## Materials and methods

### Study design and setting

This cross-sectional study was conducted on the family members of ICU patients in the Yueyang Hospital of Integrated Traditional Chinese and Western Medicine, affiliated with Shanghai University of Traditional Chinese Medicine, between July 1, 2025, and March 31, 2026.

### Study participants and sampling

Eligible participants were required to meet the following criteria: (1) being an immediate family member of an ICU patient receiving invasive mechanical ventilation; (2) being aged 18 years or older; (3) participating in the patient’s daily care; and (4) being able to understand the purpose of the study and voluntarily sign the informed consent form. Participants were excluded if they withdrew consent or submitted questionnaires that did not meet the predefined quality control criteria.

Convenience sampling was used in this study. Eligible family members were approached during ICU visits after the patient had commenced invasive mechanical ventilation. Recruitment was conducted primarily during daytime shifts, and more than one eligible family member per patient could participate. The study purpose, questionnaire procedures, and voluntary nature of participation were explained, and informed consent was obtained before questionnaire completion. Questionnaires were administered through both online electronic forms and offline paper-based forms, with the ICU family reception room serving as the main site for questionnaire distribution. Before data entry, all paper questionnaires were reviewed, and any questionable responses were verified. Data were entered twice by the same researcher and cross-checked to ensure accuracy.

To ensure data quality and enhance the credibility of the findings, predefined questionnaire screening criteria were applied to identify and exclude invalid responses or questionnaires with potential information bias: (1) an attention-check item based on common knowledge was included to identify respondents who did not read the questions carefully or answered randomly; (2) responses with obvious numerical abnormalities, such as implausible age values, were excluded to reduce the influence of data entry errors and outliers; (3) questionnaires showing repetitive response patterns, such as selecting the same option for all items, were considered invalid because they suggested non-genuine responses; and (4) questionnaires with logical inconsistencies in multiple-choice items were excluded, such as selecting one or more specific sources of information about ICU ventilator use and VAP while also selecting “none of the above.”

The sample size was estimated according to the commonly used rule of thumb for structural equation modeling (SEM), which recommends at least 5–10 participants per observed variable (16, 17). In this study, the questionnaire included 29 KAP items, consisting of 12 knowledge items, 10 attitude items, and 7 practice items. Therefore, the minimum required sample size was estimated to be 145–290 participants. Considering potential invalid questionnaires and incomplete responses, the target sample size was increased accordingly. A total of 460 questionnaires were collected, and 406 valid responses were included in the final analysis, which exceeded the minimum requirement for SEM.

### Data collection tool and technique

The questionnaire was developed with reference to previous literature and relevant guidelines, including the *Chinese Guidelines for the Diagnosis and Treatment of Hospital-Acquired Pneumonia and Ventilator-Associated Pneumonia in Adults* (18) and *Strategies to Prevent Ventilator-Associated Pneumonia, Ventilator-Associated Events, and Non-Ventilator Hospital-Acquired Pneumonia in Acute-Care Hospitals: 2022 Update* (19). After the initial draft was prepared, feedback was obtained from three ICU nursing experts, each with more than 20 years of clinical experience, and the questionnaire was revised accordingly. Based on their comments, the comprehensibility and practical applicability of the questionnaire were improved by refining items related to caregiving time, payment methods, occupational classification, and medical history, as well as by simplifying medical terminology. Practice-related items were also adjusted to better reflect the actual roles of family members of ICU patients. Items that were difficult for respondents to judge or perform were removed, whereas actionable behaviors, such as wearing isolation gowns and promptly informing healthcare staff of ventilator alarms, were retained. In addition, attention-check items were incorporated to enhance response validity. A pilot survey involving 43 participants was subsequently conducted, followed by reliability testing. The overall Cronbach’s α coefficient was 0.927, with values of 0.947, 0.905, and 0.851 for the knowledge, attitude, and practice dimensions, respectively, indicating good internal consistency for both the full questionnaire and its individual dimensions.

The final Chinese-language questionnaire consisted of four sections: demographic information, knowledge, attitudes, and practices. The demographic section collected information on family members’ age, gender, education level, place of residence, occupation, marital status, monthly income, relationship with the patient, primary caregiver status, and average caregiving time, as well as the patient’s gender, age, duration of mechanical ventilation, and comorbidities. The knowledge dimension comprised 12 items, with responses of “very familiar,” “have heard of it,” and “not clear” scored as 2, 1, and 0 points, respectively; the total score ranged from 0 to 24. The attitude dimension included 10 items assessed using a five-point Likert scale, ranging from 5 for “strongly agree” to 1 for “strongly disagree,” with a total score ranging from 10 to 50. The practice dimension contained 7 items, scored from 1 for “never” to 5 for “always,” yielding a total score ranging from 7 to 35. Thresholds for good knowledge, positive attitudes, and proactive practices were defined as scores reaching at least 70.0% of the corresponding total score, in line with criteria commonly applied in previous KAP studies (20, 21).

### Statistical analysis

Statistical analysis was conducted using SPSS version 26.0 (IBM, Armonk, NY, USA) and AMOS version 24.0 (IBM, Armonk, NY, USA). Continuous variables are presented as means and standard deviations. Categorical variables are reported as n (%). Continuous variables were assessed for normality. For comparisons between two groups, the independent-samples t-test was used for normally distributed continuous variables, whereas the Wilcoxon Mann–Whitney test was applied for non-normally distributed variables. For comparisons among three or more groups, one-way analysis of variance was used when the data were normally distributed and showed homogeneity of variance; otherwise, the Kruskal–Wallis test was performed. Correlations among knowledge, attitude, and practice scores were examined using Spearman correlation analysis. Each knowledge, attitude, and practice score was further converted into a binary outcome according to the median value of the corresponding dimension. Univariate and multivariate logistic regression analyses were then performed to identify independent influencing factors for each KAP dimension. Based on the KAP theoretical framework, SEM was used to examine the relationships among knowledge, attitude, and practice, with particular attention to the potential mediating role of attitude between knowledge and practice. Direct and indirect associations were estimated and compared. Model fit was evaluated using the root mean square error of approximation (RMSEA), standardized root mean square residual (SRMR), Tucker–Lewis index (TLI), and comparative fit index (CFI). Acceptable model fit was defined as RMSEA < 0.08, SRMR < 0.08, TLI > 0.80, and CFI > 0.80. A two-sided *p*-value less than 0.05 was considered statistically significant.

## Results

### Demographic information on participants and KAP scores

Initially, this study collected a total of 460 questionnaires. The following questionnaires were excluded: 44 with failed attention-check responses identified during quality control, 4 with implausible age values, and 6 with logical inconsistencies in multiple-choice responses. Finally, 406 valid questionnaires were retained, yielding a validity rate of 88.26%. The sample was predominantly female (56.9%), with a mean age distribution across <40 years (24.1%), 40–49 years (26.8%), 50–59 years (30.5%), and ≥60 years (18.5%). Most participants resided in urban areas (81.5%), had fixed employment (59.6%), and were married (67.7%). Regarding sources of information about ICU ventilator use and VAP, the majority (81.53%) reported none of the listed sources, followed by health education from hospitals or medical staff (17.24%), social media (7.64%), communication with relatives, friends, or other patients (7.14%), and traditional media (2.22%).

The mean knowledge, attitude, and practice scores were 3.48 ± 5.26 (possible range: 0–24), 38.94 ± 6.62 (possible range: 10–50), and 19.33 ± 6.50 (possible range: 7–35), respectively. Knowledge scores differed significantly by average monthly income per capita, relationship to the patient, and patient’s age (all *p* < 0.05). Attitude scores varied significantly with occupation type, relationship to the patient, primary caregiver status, average weekly caregiving time, and patient’s age (all *p* < 0.05). Practice scores showed significant associations with education level, relationship to the patient, primary caregiver status, and average weekly caregiving time (all *p* < 0.05) (Table 1).

**Table 1:** Baseline characteristics and KAP scores of included participants.

*N* = 406	*N* (%)	Knowledge	*p*	Attitude	*p*	Practice	*p*
		mean (SD)		mean (SD)		mean (SD)	
Total score	406 (100.0)	3.48 (5.26)		38.94 (6.62)		19.33 (6.50)	
Gender			0.846		0.830		0.752
Male	175 (43.1)	3.62 (5.53)		38.90 (6.35)		19.35 (6.77)	
Female	231 (56.9)	3.37 (5.06)		38.97 (6.84)		19.32 (6.30)	
Age (years)			0.543		0.166		0.336
<40	98 (24.1)	3.89 (5.59)		38.32 (6.64)		20.02 (6.98)	
40–49	109 (26.8)	3.35 (5.34)		38.36 (6.80)		18.66 (6.11)	
50–59	124 (30.5)	2.84 (4.49)		38.93 (6.19)		18.68 (5.90)	
≥60	75 (18.5)	4.19 (5.82)		40.60 (6.88)		20.51 (7.19)	
Place of residence			0.233		0.154		0.216
Rural	75 (18.5)	3.76 (4.77)		37.87 (7.41)		18.77 (7.24)	
Urban	331 (81.5)	3.41 (5.37)		39.18 (6.42)		19.46 (6.33)	
Education level			0.567		0.219		0.034
Junior high school or below	135 (33.3)	2.90 (4.75)		39.92 (6.55)		18.99 (6.97)	
High school/technical secondary school	76 (18.7)	2.95 (4.44)		38.21 (7.13)		19.26 (6.21)	
Associate degree	82 (20.2)	3.40 (4.51)		37.84 (5.87)		18.27 (5.74)	
Bachelor’s degree or above	113 (27.8)	4.58 (6.58)		39.04 (6.78)		20.58 (6.52)	
Occupation type			0.755		0.004		0.980
Fixed employment	242 (59.6)	3.43 (5.23)		38.12 (6.79)		19.14 (6.02)	
Non-fixed employment (e.g., freelancer, homemaker, retired)	164 (40.4)	3.55 (5.32)		40.14 (6.19)		19.63 (7.16)	
Marital status			0.561		0.343		0.194
Married	275 (67.7)	3.51 (5.51)		39.16 (6.40)		19.58 (6.49)	
Other	131 (32.3)	3.41 (4.71)		38.47 (7.07)		18.82 (6.52)	
Average monthly income per capita (Yuan)			0.015		0.117		0.657
<5,000	133 (32.8)	2.63 (4.65)		39.77 (6.48)		19.05 (6.84)	
5,000–10,000	155 (38.2)	3.21 (4.73)		38.94 (6.77)		19.65 (6.64)	
10,000–20,000	93 (22.9)	4.99 (6.48)		37.86 (6.53)		19.39 (5.93)	
>20,000	25 (6.2)	4.00 (5.50)		38.48 (6.58)		18.72 (6.03)	
Relationship to the patient			0.022		0.002		0.002
Spouse	82 (20.2)	3.91 (4.95)		41.09 (6.35)		21.51 (7.15)	
Child	215 (53.0)	2.81 (4.76)		38.14 (6.49)		19.07 (5.53)	
Other relative	109 (26.8)	4.46 (6.21)		38.88 (6.78)		18.23 (7.38)	
Primary caregiver			0.640		0.002		0.001
No	180 (44.3)	3.33 (5.38)		37.88 (6.50)		18.18 (6.45)	
Yes	226 (55.7)	3.60 (5.17)		39.77 (6.62)		20.25 (6.41)	
Average weekly caregiving time			0.060		0.015		0.001
<30 min	131 (32.3)	2.81 (4.78)		37.50 (6.53)		17.56 (6.10)	
30 min–2 h	134 (33.0)	3.90 (5.17)		39.49 (5.90)		19.56 (5.80)	
>2 h	141 (34.7)	3.70 (5.73)		39.74 (7.16)		20.77 (7.12)	
Patient’s gender			0.427		0.946		0.952
Male	235 (57.9)	3.38 (5.33)		39.04 (6.83)		19.40 (6.83)	
Female	171 (42.1)	3.61 (5.18)		38.79 (6.34)		19.25 (6.03)	
Patient’s age (years)			0.034		0.040		0.087
<60	70 (17.2)	4.80 (5.52)		40.69 (6.00)		19.94 (7.14)	
60–69	89 (21.9)	4.09 (5.67)		39.02 (6.66)		20.57 (7.50)	
70–79	176 (43.3)	2.85 (4.69)		38.01 (6.72)		18.35 (5.87)	
≥80	71 (17.5)	2.96 (5.59)		39.41 (6.64)		19.62 (5.68)	
Duration of ventilator use			0.383		0.697		0.144
≤3 days	181 (44.6)	3.71 (5.50)		38.90 (6.52)		19.55 (6.70)	
4–7 days	112 (27.6)	2.44 (3.66)		38.53 (6.30)		18.13 (5.43)	
>7 days	113 (27.8)	4.13 (6.05)		39.41 (7.11)		20.18 (7.02)	
Current payment methods			0.567		0.314		0.553
Fully covered by medical insurance	151 (37.2)	3.60 (5.51)		38.70 (6.70)		19.50 (6.24)	
Partially self-paid and partially covered by medical insurance	255 (62.8)	3.40 (5.12)		39.07 (6.59)		19.24 (6.66)	

### Distribution of responses to knowledge, attitude, and practice

For the knowledge dimension, the proportion of “not clear” responses exceeded 65% across all items, indicating broad knowledge gaps regarding VAP prevention. The highest unfamiliarity was observed for improper suctioning procedures increasing pneumonia risk (K5, 83.25%), the definition of VAP as pneumonia occurring after more than 48 h of mechanical ventilation (K1, 83.00%), typical VAP symptoms such as fever, increased sputum production, and dyspnea (K11, 82.51%), head-of-bed elevation for reducing reflux and infection risk (K9, 79.06%), oral care for pneumonia prevention (K8, 78.57%), and the risk associated with prolonged supine positioning (K3, 77.83%) (Supplementary Table S1).

For the attitude dimension, participants generally showed positive attitudes, but relatively weaker willingness was observed for cooperating with healthcare staff in oral care (A4: 60.10% agreed, 21.43% neutral, and 18.47% disagreed), participating in daily care such as repositioning to prevent infection (A3: 62.06% agreed, 18.97% neutral, and 18.97% disagreed), and learning more about VAP-related knowledge (A10: 62.56% agreed, 23.89% neutral, and 13.55% disagreed) (Supplementary Table S2).

For the practice dimension, the most prominent gap was active information seeking, with only 15.76% of participants reporting that they always or often asked healthcare staff how to prevent VAP, while 42.12% never did so (P7). Other suboptimal practices included hand hygiene before visiting the patient (P1: 27.83% always/often; 13.79% never), observation of worsening symptoms such as increased coughing (P3: 32.52%; 10.34%), assistance with oral care (P4: 27.83%; 20.69%), repositioning or head-of-bed elevation (P5: 29.31%; 17.98%), and encouraging coughing exercises when the patient was conscious (P6: 32.76%; 20.94%) (Supplementary Table S3).

### Correlations between KAP

Spearman correlation analysis showed that both Knowledge (r = 0.204, *p* < 0.001) and Attitude (r = 0.356, *p* < 0.001) were positively correlated with Practice. No significant correlation was found between Knowledge and Attitude (*p* = 0.154) (Supplementary Table S4).

### Factors associated with KAP

In the multivariate logistic regression analysis, a monthly per capita income of 10,000–20,000 RMB (OR = 2.118, 95% CI: [1.217–3.689], *p* = 0.008) and an average weekly caregiving time of 30 min to 2 h (OR = 1.701, 95% CI: [1.034–2.798], *p* = 0.037) were independently associated with better knowledge. Meanwhile, being the child of the patient (vs. spouse) (OR = 0.451, 95% CI: [0.208–0.975], *p* = 0.043) was independently associated with a less positive attitude, whereas being the primary caregiver (OR = 1.781, 95% CI: [1.066–2.974], *p* = 0.027) was independently associated with a more positive attitude. Further, higher knowledge scores (OR = 1.111, 95% CI: [1.057–1.168], *p* < 0.001) and higher attitude scores (OR = 1.122, 95% CI: [1.079–1.166], *p* < 0.001) were independently associated with better practice. Being aged 40–49 years (OR = 0.389, 95% CI: [0.195–0.776], *p* = 0.007) or ≥60 years (OR = 0.374, 95% CI: [0.150–0.934], *p* = 0.035), and being an “other relative” of the patient (vs. spouse) (OR = 0.403, 95% CI: [0.168–0.968], *p* = 0.042) were independently associated with poorer practice (Tables 2–4).

**Table 2:** Univariate and multivariate analysis of factors associated with knowledge scores.

Knowledge	Univariate analysis	*p*	Multivariate analysis	*p*
	OR (95%CI)		OR (95%CI)	
Gender				
Male				
Female	0.98 (0.66, 1.45)	0.915		
Age				
<40				
40–49	0.89 (0.52, 1.55)	0.686		
50–59	1.04 (0.61, 1.76)	0.899		
≥60	1.28 (0.70, 2.34)	0.428		
Place of residence				
Rural				
Urban	0.72 (0.43, 1.18)	0.193		
Education level				
Junior high school or below				
High school/technical secondary school	1.09 (0.62, 1.92)	0.76		
Associate degree	1.05 (0.60, 1.82)	0.868		
Bachelor’s degree or above	1.11 (0.67, 1.84)	0.682		
Occupation type				
Fixed employment				
Non-fixed employment (e.g., freelancer, homemaker, retired)	1.07 (0.72, 1.59)	0.737		
Marital status				
Married				
Other	1.20 (0.79, 1.82)	0.393		
Average monthly income per capita				
<5, 000				
5, 000–10, 000	1.40 (0.87, 2.24)	0.166	1.38 (0.85, 2.22)	0.193
10, 000–20, 000	2.23 (1.30, 3.84)	0.004	2.12 (1.22, 3.69)	0.008
>20, 000	1.48 (0.62, 3.52)	0.368	1.47 (0.61, 3.52)	0.393
Relationship to the patient				
Spouse				
Child	0.66 (0.40, 1.10)	0.111		
Other relative	1.12 (0.63, 2.00)	0.69		
Primary caregiver				
No				
Yes	0.99 (0.67, 1.47)	0.967		
Average weekly caregiving time				
<30 min				
30 min–2 h	1.87 (1.15, 3.05)	0.012	1.70 (1.03, 2.80)	0.037
>2 h	1.03 (0.63, 1.66)	0.917	0.99 (0.60, 1.64)	0.981
Patient’s gender				
Male				
Female	1.20 (0.81, 1.78)	0.376		
Patient’s age				
<60				
60–69	0.81 (0.43, 1.52)	0.518	0.86 (0.45, 1.63)	0.642
70–79	0.58 (0.33, 1.01)	0.053	0.60 (0.34, 1.06)	0.08
≥80	0.62 (0.32, 1.20)	0.153	0.60 (0.30, 1.20)	0.147
Duration of ventilator use				
<3 days				
4–7 days	0.87 (0.54, 1.40)	0.568		
>7 days	0.92 (0.57, 1.48)	0.732		
Current payment methods				
Fully covered by medical insurance				
Partially self-paid and partially covered by medical insurance	0.82 (0.55, 1.23)	0.333		

**Table 3:** Univariate and multivariate analysis of factors associated with attitude.

Attitude	Univariate analysis	*p*	Multivariate analysis	*p*
	OR (95%CI)		OR (95%CI)	
Knowledge	1.02 (0.99, 1.06)	0.223	1.03 (0.99, 1.07)	0.204
Gender				
Male				
Female	0.83 (0.56, 1.23)	0.354		
Age				
<40				
40–49	1.33 (0.77, 2.30)	0.309	1.45 (0.79, 2.70)	0.234
50–59	0.99 (0.58, 1.68)	0.955	0.72 (0.38, 1.34)	0.296
≥60	1.72 (0.94, 3.19)	0.081	0.66 (0.29, 1.52)	0.331
Place of residence				
Rural				
Urban	1.31 (0.80, 2.18)	0.287		
Education level				
Junior high school or below				
High school/technical secondary school	0.72 (0.41, 1.26)	0.243		
Associate degree	0.72 (0.41, 1.24)	0.237		
Bachelor’s degree or above	0.79 (0.48, 1.31)	0.369		
Occupation type				
Fixed employment				
Non-fixed employment (e.g., freelancer, homemaker, retired)	1.74 (1.17, 2.61)	0.007	1.59 (0.94, 2.69)	0.081
Marital status				
Married				
Other	0.68 (0.45, 1.04)	0.075	0.82 (0.51, 1.33)	0.425
Average monthly income per capita				
<5,000				
5,000–10,000	0.65 (0.41, 1.04)	0.072	0.72 (0.44, 1.20)	0.208
10,000–20,000	0.55 (0.32, 0.94)	0.03	0.63 (0.34, 1.15)	0.131
>20,000	0.43 (0.17, 1.01)	0.057	0.49 (0.19, 1.25)	0.134
Relationship to the patient				
Spouse				
Child	0.38 (0.22, 0.65)	0.001	0.45 (0.21, 0.98)	0.043
Other relative	0.45 (0.24, 0.81)	0.009	0.61 (0.27, 1.38)	0.232
Primary caregiver				
No				
Yes	1.98 (1.34, 2.96)	0.001	1.78 (1.07, 2.97)	0.027
Average weekly caregiving time				
<30 min				
30 min–2 h	1.02 (0.63, 1.65)	0.944	0.80 (0.45, 1.44)	0.464
>2 h	1.74 (1.08, 2.83)	0.024	0.96 (0.50, 1.83)	0.899
Patient’s gender				
Male				
Female	1.03 (0.69, 1.53)	0.887		
Patient’s age				
<60				
60–69	0.70 (0.37, 1.32)	0.277	0.75 (0.36, 1.56)	0.442
70–79	0.57 (0.32, 1.00)	0.054	0.92 (0.45, 1.88)	0.817
≥80	0.72 (0.37, 1.41)	0.343	1.44 (0.60, 3.49)	0.418
Duration of ventilator use				
<3 days				
4–7 days	0.91 (0.56, 1.45)	0.679		
>7 days	1.06 (0.66, 1.70)	0.803		
Current payment methods				
Fully covered by medical insurance				
Partially self-paid and partially covered by medical insurance	1.34 (0.89, 2.00)	0.16		

**Table 4:** Univariate and multivariate analysis of factors associated with practice.

Practice	Univariate analysis	*p*	Multivariate analysis	*p*
	OR (95%CI)		OR (95%CI)	
Knowledge	1.10 (1.06, 1.15)	<0.001	1.11 (1.06, 1.17)	<0.001
Attitude	1.12 (1.08, 1.16)	<0.001	1.12 (1.08, 1.17)	<0.001
Gender				
Male				
Female	1.30 (0.88, 1.93)	0.194		
Age				
<40				
40–49	0.53 (0.31, 0.92)	0.026	0.39 (0.20, 0.78)	0.007
50–59	0.70 (0.41, 1.19)	0.187	0.53 (0.26, 1.08)	0.081
≥60	0.87 (0.48, 1.59)	0.656	0.37 (0.15, 0.93)	0.035
Place of residence				
Rural				
Urban	1.48 (0.89, 2.50)	0.131		
Education level				
Junior high school or below				
High school/technical secondary school	1.20 (0.68, 2.10)	0.537	1.35 (0.71, 2.57)	0.358
Associate degree	0.85 (0.48, 1.48)	0.568	0.74 (0.37, 1.48)	0.398
Bachelor’s degree or above	1.67 (1.01, 2.78)	0.045	1.41 (0.75, 2.67)	0.285
Occupation type				
Fixed employment				
Non-fixed employment (e.g., freelancer, homemaker, retired)	0.95 (0.64, 1.41)	0.785		
Marital status				
Married				
Other	0.70 (0.45, 1.06)	0.09	0.69 (0.41, 1.17)	0.169
Average monthly income per capita				
<5,000				
5,000–10,000	1.15 (0.72, 1.83)	0.566		
10,000–20,000	1.13 (0.66, 1.92)	0.661		
>20,000	1.16 (0.49, 2.74)	0.737		
Relationship to the patient				
Spouse				
Child	0.60 (0.35, 1.00)	0.05	1.09 (0.48, 2.49)	0.844
Other relative	0.38 (0.21, 0.67)	0.001	0.40 (0.17, 0.97)	0.042
Primary caregiver				
No				
Yes	1.68 (1.13, 2.50)	0.011	1.28 (0.74, 2.22)	0.379
Average weekly caregiving time				
<30 min				
30 min–2 h	1.43 (0.88, 2.34)	0.148	0.76 (0.40, 1.42)	0.382
>2 h	1.73 (1.07, 2.82)	0.026	0.87 (0.44, 1.74)	0.703
Patient’s gender				
Male				
Female	0.98 (0.66, 1.45)	0.903		
Patient’s age				
<60				
60–69	0.80 (0.43, 1.51)	0.493	1.14 (0.52, 2.52)	0.74
70–79	0.44 (0.25, 0.77)	0.004	0.74 (0.34, 1.61)	0.453
≥80	0.86 (0.44, 1.68)	0.666	1.38 (0.53, 3.64)	0.512
Duration of ventilator use				
<3 days				
4–7 days	0.91 (0.57, 1.46)	0.699		
>7 days	1.03 (0.65, 1.66)	0.89		
Current payment methods				
Fully covered by medical insurance				
Partially self-paid and partially covered by medical insurance	1.01 (0.68, 1.52)	0.952		

### Interactions between KAP

The fit of the SEM model yielded good indices demonstrating good model fit (RMSEA value: 0.074, SRMR value: 0.077, TLI value: 0.900, and CFI value: 0.910) (Supplementary Table S5), and the effect estimates between KAP were detailed in Supplementary Table S6. SEM results show that knowledge had direct effects on attitude (*β* = 0.190, *p* < 0.001) and practice (*β* = 0.222, *p* < 0.001). Meanwhile, attitude had a direct effect on practice (*β* = 0.329, *p* < 0.001). Furthermore, knowledge indirectly affected practice through attitude (*β* = 0.063, *p* < 0.001) (Table 5; Figure 1).

**Table 5:** SEM analysis of direct and indirect effects.

Model paths		Total effects		Direct effect		Indirect effect	
		*β* (95%CI)	*p*	*β* (95%CI)	*p*	*β* (95%CI)	*p*
Attitude	Knowledge	0.190 (0.090, 0.291)	<0.001	0.190 (0.090, 0.291)	<0.001		
Practice	Knowledge	0.285 (0.183, 0.387)	<0.001	0.222 (0.121, 0.323)	<0.001	0.063 (0.025, 0.100)	0.001
	Attitude	0.329 (0.231, 0.426)	<0.001	0.329 (0.231, 0.426)	<0.001		

**Figure 1:**
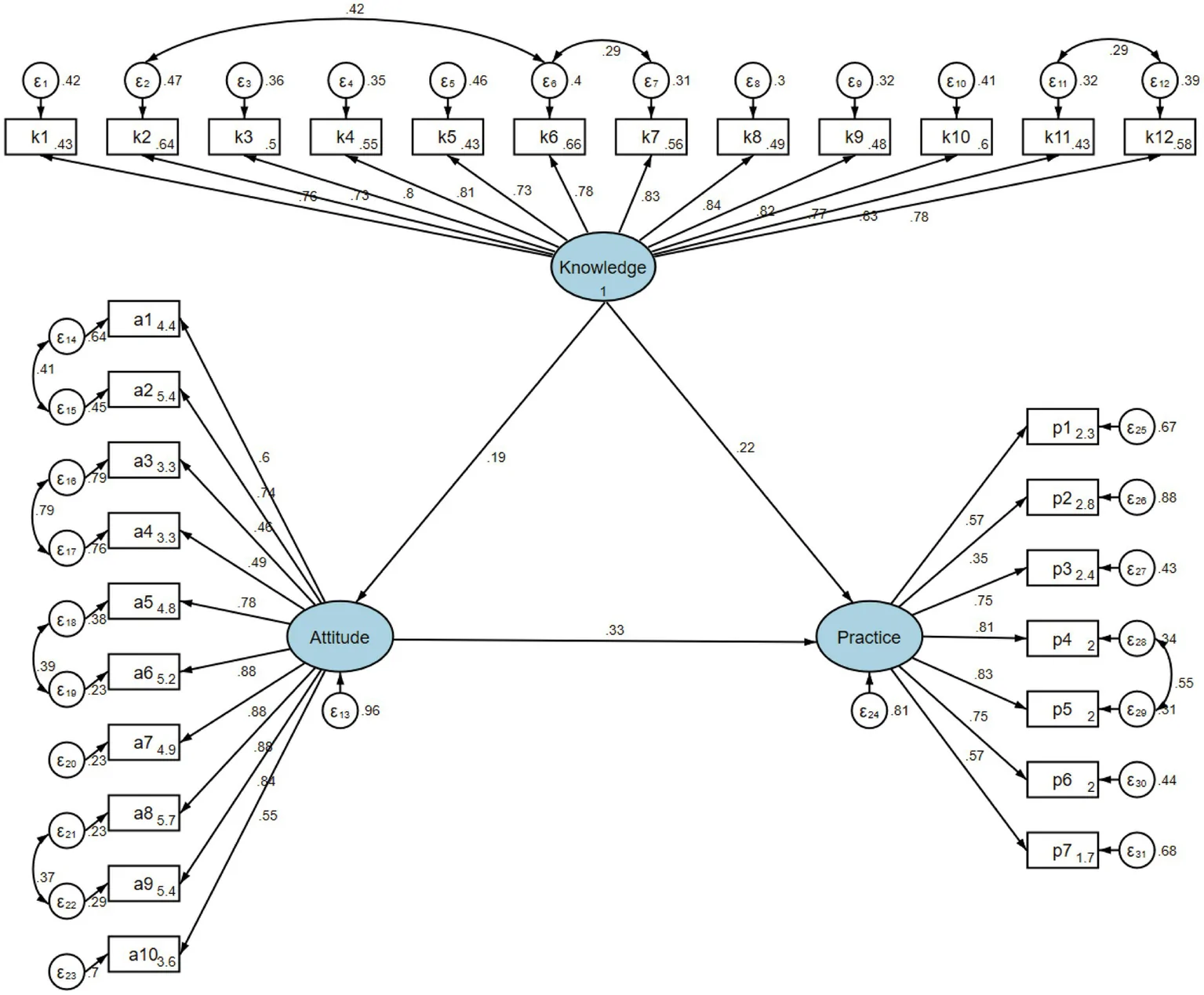
Structural equation model of the relationships among KAP regarding VAP among family members of ICU patients. Ovals represent latent constructs, and rectangles represent observed variables/items. Small circles indicate measurement errors or residual variances of observed variables. Single-headed arrows indicate hypothesized directional paths or factor loadings, while double-headed curved arrows indicate correlated residuals. Standardized path coefficients are displayed on the corresponding arrows, representing the strength of associations between constructs or factor loadings between latent constructs and observed variables. The numerical values within the rectangles represent the variances of the corresponding observed variables.

## Discussion

Family members of ICU patients demonstrated insufficient VAP-related knowledge and suboptimal preventive practices despite generally positive attitudes, suggesting that willingness to participate has not yet been effectively translated into informed bedside behaviors. ICU teams should develop structured, family-oriented VAP education and participation pathways, including brief admission-based teaching, visual bedside reminders, and supervised involvement in feasible preventive practices such as hand hygiene, symptom observation, oral-care cooperation, and appropriate communication with healthcare staff.

The overall KAP pattern observed in this study differs from the assumption that positive attitudes alone are sufficient to support preventive engagement. Related research in infection prevention has shown that informal caregivers often express strong motivation to contribute to patient care while simultaneously reporting uncertainty regarding appropriate preventive behaviors and limited access to practical instruction (22). Studies conducted in ICU settings have also noted that family members frequently receive fragmented information focused on patient updates rather than structured education regarding infection prevention and supportive care responsibilities (23). The present findings extend this concern to VAP prevention, where the gap between favorable attitudes and actual practices appeared substantial.

Knowledge deficits were particularly evident in areas directly related to common VAP-prevention measures, including oral care, head-of-bed elevation, symptom recognition, and the risks associated with inappropriate suctioning procedures. These findings are clinically important because they involve routine preventive measures that families may observe frequently during ICU visits. Previous studies have suggested that caregiver understanding of infection-prevention procedures is often limited when explanations rely heavily on technical terminology or when communication occurs during periods of emotional distress (24). Research on family-centered ICU care has also reported that relatives may hesitate to ask questions because they perceive the ICU as a highly specialized environment in which clinical authority belongs primarily to healthcare professionals (25). This may partially explain why the proportion of respondents actively seeking information from staff remained low.

The relationship between knowledge and practice identified in the present study is consistent with evidence showing that educational exposure influences adherence to infection-prevention behaviors among both professional and non-professional caregivers (26). However, the relatively weak direct association between knowledge and attitudes in the correlation analysis suggests that acquiring information alone may not substantially alter perceptions of involvement. Attitudes toward participation are likely shaped by several additional factors, including emotional burden, trust in staff, perceived competence, and uncertainty regarding acceptable caregiving boundaries. Related ICU studies have reported that family members often prefer supportive and observational roles unless staff explicitly encourage more active participation (27).

The practice findings deserve particular consideration because the least frequently performed behaviors were largely feasible supportive activities rather than technically demanding procedures. Low rates of hand hygiene before patient contact, limited participation in oral-care support, and infrequent monitoring of symptom changes suggest that family members were not fully incorporated into bedside prevention processes. Research examining family engagement in ICU care has emphasized that participation tends to remain passive when institutions do not provide structured opportunities for involvement or clear behavioral expectations (28). In many hospitals, family participation remains dependent on individual staff communication styles rather than standardized care processes. Under such conditions, preventive behaviors may occur inconsistently even when families express willingness to help.

Differences observed across caregiver roles also provide insight into how participation develops within ICU settings. Primary caregivers showed more favorable attitudes toward prevention, while children of patients demonstrated less positive attitudes compared with spouses. Previous studies have suggested that caregiving identity strongly influences engagement in hospital care, particularly when prolonged illness creates sustained caregiving responsibilities outside the ICU environment (29). Spouses and primary caregivers may therefore perceive bedside participation as a continuation of existing caregiving duties, whereas other relatives may view ICU care as primarily the responsibility of healthcare professionals. These differences are relevant because the SEM findings indicate that attitudes contributed directly to practice outcomes.

The association between caregiving exposure and knowledge may also reflect differences in communication opportunities. Participants with moderate caregiving time demonstrated better knowledge levels, whereas prolonged caregiving exposure did not independently predict improved knowledge after adjustment. This suggests that repeated ICU exposure does not necessarily produce learning in the absence of structured communication. Related research on health literacy in acute-care settings has shown that families often struggle to retain clinical information when communication occurs informally or during emotionally stressful encounters (29). Educational interventions that rely solely on spontaneous bedside explanations may therefore be insufficient.

The absence of clear differences across several demographic variables suggests that inadequate VAP-related knowledge and preventive behavior are not restricted to a narrow subgroup of family members. Similar observations have been reported in studies examining infection-prevention awareness among hospital visitors and caregivers, where poor adherence to preventive measures was observed across diverse demographic categories (29). This pattern indicates that the problem may be more closely related to institutional communication practices than to individual social characteristics. ICU infection prevention has traditionally focused on healthcare staff adherence to ventilator bundles and environmental control measures, while family participation has received comparatively limited attention (30).

Family involvement varies across ICU settings; therefore, reported practice frequencies should be interpreted in light of opportunities for participation and may not solely reflect family members’ knowledge or willingness. The findings support several practical changes in ICU care. Educational interventions for family members should focus on specific preventive behaviors that families can realistically perform or support. Brief nurse-led teaching sessions during the early stages of mechanical ventilation may be more effective than distributing written information alone. Studies evaluating teach-back approaches in hospital settings have reported improved caregiver understanding and recall when family members are asked to repeat instructions in their own words (29). In the context of VAP prevention, this approach may help clarify misconceptions regarding hand hygiene, oral care, positioning, and symptom monitoring.

ICUs may also benefit from introducing structured participation protocols that define which preventive activities are appropriate for family involvement and which remain exclusively clinical responsibilities. Research on family-centered critical care has suggested that clearly defined participation frameworks can improve communication and reduce uncertainty among relatives (31). For example, family members could receive simple bedside guidance regarding hand hygiene procedures, observation of secretion changes, or when to notify staff about ventilator alarms or worsening respiratory symptoms. Without explicit guidance, families may avoid participation because of concern about interfering with treatment.

Staff training is equally important. Healthcare professionals may underestimate the amount of practical explanation required for family participation in infection prevention. Communication strategies should therefore emphasize concise explanations, repetition of key preventive actions, and consistent terminology across staff members. Educational materials alone are unlikely to be sufficient if they are not reinforced during daily interactions. Digital tools such as short videos or QR-code educational modules may provide additional support, although previous studies indicate that such interventions are most effective when integrated into direct communication rather than replacing it entirely (32).

This study has several limitations. First, the single-center cross-sectional design may limit the external generalizability of the findings and precludes causal inference regarding the relationships among knowledge, attitudes, and practices. Second, the use of convenience sampling and self-reported questionnaire data may have introduced selection bias, recall bias, or social desirability bias, although questionnaire quality control procedures and multivariable analyses were used to reduce potential information bias and confounding. Third, family members’ actual participation in VAP prevention was not objectively observed, which may have led to discrepancies between reported and real-world practices. Fourth, more than one family member per patient could participate. Because questionnaires were anonymous and no patient linkage identifiers were recorded, within-patient clustering could not be assessed or accounted for, potentially affecting the precision of the estimates.

## Conclusion

Overall, family members of ICU patients demonstrated insufficient VAP-related knowledge and suboptimal preventive practices despite generally positive attitudes, and the observed knowledge–attitude–practice pathway suggests that improving understanding and strengthening willingness to participate may promote safer bedside behaviors. Clinically, ICU teams should implement structured, family-oriented VAP prevention education combined with feasible bedside participation protocols, such as supervised hand hygiene, symptom observation, oral-care support, and timely communication about ventilator-related concerns. Future multicenter longitudinal studies are warranted to further validate these findings and determine whether structured family education and participation programs can improve preventive behaviors and ultimately contribute to better patient outcomes.

## Data Availability

The original contributions presented in the study are included in the article/Supplementary material, further inquiries can be directed to the corresponding author.
