# Supplementary material for: Knowledge, attitudes, and practices regarding ventilator-associated pneumonia among family members of ICU patients
Source: Front Public Health. 2026 Oct 30;14:1997727. doi: 10.3389/fpubh.2026.1997727 (PMC13759480; doi:10.3389/fpubh.2026.1997727)
Supplement: Supplementary file 1 [file Table_1.docx]

**Supplementary table 1. Distribution of knowledge dimension responses**

| **Knowledge items, n (%)** | Very familiar | Have heard of it | Not clear |
| --- | --- | --- | --- |
| 1. “Ventilator-associated pneumonia (VAP)” refers to a lung infection that occurs after a patient has been on mechanical ventilation for more than 48 hours. | 10 (2.46%) | 59 (14.53%) | 337 (83%) |
| 2. Developing VAP can prolong hospital stay and increase medical costs. | 27 (6.65%) | 105 (25.86%) | 274 (67.49%) |
| 3. Prolonged supine positioning increases the risk of pneumonia. | 19 (4.68%) | 71 (17.49%) | 316 (77.83%) |
| 4. Long-term use of sedative medications can prolong the duration of mechanical ventilation, thereby increasing the risk of pneumonia. | 13 (3.2%) | 89 (21.92%) | 304 (74.88%) |
| 5. Improper suctioning procedures (e.g., excessive frequency or poor hygiene) can increase the risk of pneumonia. | 8 (1.97%) | 60 (14.78%) | 338 (83.25%) |
| 6. The nutritional status of ICU patients is closely related to immunity, and malnutrition increases the risk of infection. | 33 (8.13%) | 104 (25.62%) | 269 (66.26%) |
| 7. Good hand hygiene practices (e.g., handwashing and disinfection) help prevent pneumonia. | 29 (7.14%) | 80 (19.7%) | 297 (73.15%) |
| 8. Regular oral care (e.g., toothbrushing or use of antibacterial mouthwash) helps prevent pneumonia. | 16 (3.94%) | 71 (17.49%) | 319 (78.57%) |
| 9. Elevating the head of the bed (30–45°) can reduce gastric reflux and lower the risk of infection. | 16 (3.94%) | 69 (17%) | 321 (79.06%) |
| 10. Early discontinuation of mechanical ventilation can reduce the risk of pneumonia. | 29 (7.14%) | 92 (22.66%) | 285 (70.2%) |
| 11. VAP may present with symptoms such as fever, increased sputum production, and dyspnea. | 12 (2.96%) | 59 (14.53%) | 335 (82.51%) |
| 12. Treatment of VAP requires the selection of appropriate antibiotics based on examination results. | 16 (3.94%) | 97 (23.89%) | 293 (72.17%) |

**Supplementary table 2. Distribution of attitude dimension responses**

| **Attitude items, n (%)** | Strongly agree | Agree | Neutral | Disagree | Strongly disagree |
| --- | --- | --- | --- | --- | --- |
| 1. I think it is important for family members to understand VAP-related knowledge. | 95 (23.4%) | 197 (48.52%) | 83 (20.44%) | 27 (6.65%) | 4 (0.99%) |
| 2. I believe that the longer the duration of mechanical ventilation, the higher the risk. | 100 (24.63%) | 213 (52.46%) | 84 (20.69%) | 9 (2.22%) | 0 (0%) |
| 3. I am willing to participate in the patient’s daily care (e.g., assisting with repositioning) to prevent infection. | 80 (19.7%) | 172 (42.36%) | 77 (18.97%) | 60 (14.78%) | 17 (4.19%) |
| 4. I am willing to cooperate with healthcare staff in performing oral care for the patient. | 86 (21.18%) | 158 (38.92%) | 87 (21.43%) | 61 (15.02%) | 14 (3.45%) |
| 5. I believe that healthcare staff are capable of effectively preventing infections. | 117 (28.82%) | 199 (49.01%) | 68 (16.75%) | 19 (4.68%) | 3 (0.74%) |
| 6. I hope healthcare staff will communicate more with us about infection risks and precautions. | 122 (30.05%) | 202 (49.75%) | 68 (16.75%) | 13 (3.2%) | 1 (0.25%) |
| 7. I am concerned that the patient’s condition may worsen due to pneumonia. | 110 (27.09%) | 203 (50%) | 74 (18.23%) | 17 (4.19%) | 2 (0.49%) |
| 8. I support daily evaluation by doctors to discontinue mechanical ventilation as early as possible. | 119 (29.31%) | 214 (52.71%) | 65 (16.01%) | 8 (1.97%) | 0 (0%) |
| 9. I am willing to actively learn about the patient’s condition and cooperate with care. | 121 (29.8%) | 214 (52.71%) | 59 (14.53%) | 11 (2.71%) | 1 (0.25%) |
| 10. I am willing to learn more about VAP-related knowledge. | 80 (19.7%) | 174 (42.86%) | 97 (23.89%) | 41 (10.1%) | 14 (3.45%) |

**Supplementary table 3. Distribution of practice dimension responses**

| **Practice items, n (%)** | Always | Often | Sometimes | Rarely | Never |
| --- | --- | --- | --- | --- | --- |
| 1. I wash and disinfect my hands carefully before visiting the patient. | 30 (7.39%) | 83 (20.44%) | 76 (18.72%) | 161 (39.66%) | 56 (13.79%) |
| 2. I try to avoid touching the ventilator tubing while accompanying the patient. | 149 (36.7%) | 91 (22.41%) | 71 (17.49%) | 68 (16.75%) | 27 (6.65%) |
| 3. I observe whether the patient has worsening symptoms such as increased coughing. | 50 (12.32%) | 82 (20.2%) | 79 (19.46%) | 153 (37.68%) | 42 (10.34%) |
| 4. I assist healthcare staff in performing oral care for the patient. | 54 (13.3%) | 59 (14.53%) | 68 (16.75%) | 141 (34.73%) | 84 (20.69%) |
| 5. I help reposition the patient or elevate the head of the bed. | 62 (15.27%) | 57 (14.04%) | 51 (12.56%) | 163 (40.15%) | 73 (17.98%) |
| 6. When the patient is conscious, I encourage them to practice coughing as instructed by the doctor. | 70 (17.24%) | 63 (15.52%) | 50 (12.32%) | 138 (33.99%) | 85 (20.94%) |
| 7. I actively ask healthcare staff how to prevent ventilator-associated pneumonia. | 34 (8.37%) | 30 (7.39%) | 32 (7.88%) | 139 (34.24%) | 171 (42.12%) |

**Supplementary table 4. Correlation analysis**

| **Spearman** | **Knowledge** | **Attitude** | **Practice** |
| --- | --- | --- | --- |
| **Knowledge** | 1.000 |  |  |
| **Attitude** | 0.071 (P=0.154) | 1.000 |  |
| **Practice** | 0.204 (P<0.001) | 0.356 (P<0.001) | 1.000 |

**Supplementary table 5. SEM fit indicators**

| **Indicators** | **Reference** | **Results** |
| --- | --- | --- |
| RMSEA | <0.080 | 0.074 |
| SRMR | <0.080 | 0.077 |
| TLI | >0.800 | 0.900 |
| CFI | >0.800 | 0.910 |

**Supplementary table 6. Estimated total effect coefficient**

| **Indicators** |  | **Estimate** | **P>\|z\|** |
| --- | --- | --- | --- |
| Attitude |  |  |  |
|  | Knowledge | 3.73 | <0.001 |
| Practice |  |  |  |
|  | Knowledge | 5.48 | <0.001 |
|  | Attitude | 6.60 | <0.001 |
